# Predictors of unmet need for contraception among adolescent girls and young women in selected high fertility countries in sub-Saharan Africa: A multilevel mixed effects analysis

**DOI:** 10.1371/journal.pone.0236352

**Published:** 2020-08-06

**Authors:** Bright Opoku Ahinkorah

**Affiliations:** The Australian Centre for Population and Public Health Research [ACPPHR], Faculty of Health, University of Technology Sydney, Liverpool, Australia; Liverpool School of Tropical Medicine, UNITED KINGDOM

## Abstract

**Introduction:**

Despite the desire of adolescent girls and young women (AGYW) in sub-Saharan Africa (SSA) to use contraceptives, the majority of them have challenges with access to contraceptive services. This is more evident in high fertility countries in SSA. The purpose of this study was to examine the predictors of unmet need for contraception among AGYW in selected high fertility countries in SSA.

**Materials and methods:**

Data from current Demographic and Health Surveys (DHS) carried out between 2010 and 2018 in 10 countries in SSA were analysed. A sample size of 24,898 AGYW who were either married or cohabiting was used. Unmet need for contraception was the outcome variable in this study. The explanatory variables were age, marital status, occupation, educational level, frequency of reading newspaper/magazine, frequency of listening to radio, frequency of watching television and parity (individual level variables) and wealth quintile, sex of household head, place of residence and decision-maker in healthcare (household/community level variables). Descriptive and multilevel logistic regression analyses were carried out. The results of the multilevel logistic regression analyses were reported using adjusted odds ratios at 95% confidence interval.

**Results:**

The prevalence of unmet need for contraception in all the countries considered in this study was 24.9%, with Angola, recording the highest prevalence of 42.6% while Niger had the lowest prevalence of 17.8%. In terms of the individual level predictors, the likelihood of unmet need for contraception was low among AGYW aged 20–24 [aOR = 0.82; 95% CI = 0.76–0.88], those with primary [aOR = 1.22; 95% CI = 1.13–1.31] and secondary/higher levels of formal education [aOR = 1.18; 95% CI = 1.08–1.28, p < 0.001], cohabiting AGYW [aOR = 1.52; 95% CI = 1.42–1.63] and AGYW with three or more births [aOR = 3.41; 95% CI = 3.02–3.85]. At the household/community level, the odds of unmet need for contraception was highest among poorer AGYW [aOR = 1.36; 95% CI = 1.21–1.53], AGYW in female-headed households [aOR = 1.22; 95% CI = 1.13–1.33], urban AGYW [aOR = 1.21; 95% CI = 1.11–1.32] and AGYW who took healthcare decisions alone [aOR = 1.10; 95% CI = 1.01–1.21].

**Conclusion:**

This study has identified disparities in unmet need for contraception among AGYW in high fertility countries in SSA, with AGYW in Angola having the highest prevalence. Both individual and household/community level factors predicted unmet need for contraception among AGYW in this study. However, based on the ICC values, household/community level factors prevailed the individual level factors. Enhancing access to contraception among poorer AGYW, those in female-headed households, those in urban areas and those who take healthcare decisions alone by both governmental and non-governmental organisations in high fertility countries is recommended.

## Introduction

A countless number of factors pose challenges to the health and wellbeing of young people, and these factors are more predominant in females [1]. Young people are defined as those aged 10 to 19 [2]. The majority of young people (89%) live in developing countries, including SSA [3] and by 2050, it has been projected that the population of young people in SSA will reach 605 million [4]. In SSA, most young people are growing up in poor settings that are associated with high unemployment rates, rapid urbanization, often limited educational opportunities, and rapidly changing socio-cultural norms and practices [5, 6]. Apart from these general challenges, the female population has to do deal with several sexual and reproductive issues, including unintended pregnancies, high fertility and abortions [7, 8].

Globally, the use of contraceptives has been considered essential in fertility regulation [9, 10] and constitutes a major feature in the reproductive health of women, especially AGYW who have the desire to space or limit childbearing [11]. Although contraceptives have been considered effective in fertility regulation, their use among AGYW in SSA remains an issue that needs urgent attention [12–14]. Studies have indicated that despite the desire for AGYW in SSA to use contraceptives, a majority of them have challenges with access to contraceptive services [15–17]. A useful measure for of the gap between the desire for and access to contraception is the estimation of unmet need for contraception [18]. The World Health Organisation (WHO) describes women with unmet need for contraception as those who are fecund and sexually active but are not using any method of contraception, and report not wanting any more children or wanting to delay the next child [19]. This indicator is essential because it provides the basis for providing contraceptive services and indicates how a country complies with its populations’ reproductive health rights [11].

Following the 2012 London Summit on Family Planning, over 40 states the world over acknowledged that life-saving contraception constitutes an essential aspect of fundamental human rights for women [13]. Although the prevalence of unmet need for contraception among AGYW in SSA is unknown, nearly 25% of women in the subregion (i.e. about 47 million) have unmet need for contraception [20], with a majority of them being AGYW [15–17]. This unquestionably accounts for the high fertility and unsafe abortion rates among AGYW in SSA [21].

Despite several interventions to reduce fertility in SSA, including tackling unmet need for contraception, a recent report on global fertility rates by the World Bank lists 10 SSA countries as countries with fertility rates above 5.0 [22]. This rate is more than the global rate of 2.4 children per woman and the average rate of 4.8 in SSA [22]. These countries and their fertility rates are as follows: Niger (7.2), Mali (5.9), Chad (5.8), Angola (5.6), Burundi (5.6), Uganda (5.5), Nigeria (5.4), Gambia (5.3), Burkina Faso (5.3) and Mozambique (5.1) [22]. With evidence that unmet need for contraception plays a key role in high fertility [23–25], and the understanding that unmet need for contraception is predominant in AGYW in SSA [15–17], it is worthwhile to understand its predictors in these high fertility countries.

However, in SSA, studies on the predictors of unmet need for contraception among AGYW have been done in specific countries like Ethiopia [13], Senegal [26] and South Africa [17]. There has not been any study that has combined specific high fertility countries in SSA together to understand the reasons for the high unmet need for contraception in AGYW who live in those countries. Moreover, these previous studies failed to take into account the household/community-level factors and how they interact with individual level factors to predict unmet need for contraception. A multilevel approach will contribute to an understanding of both the individual and household/community level factors that predict unmet need for contraception.

Hence, this study seeks to fill this gap by examining the predictors of unmet need for contraception among AGYW in Niger, Mali, Chad, Angola, Burundi, Uganda, Nigeria, Gambia, Burkina Faso and Mozambique using a multilevel modelling. Findings from the study will help governments and non-governmental organisations to improve access to modern contraception among AGYW in high fertility countries in SSA. The findings will also form the basis for improving sexual and reproductive health policies aimed at addressing unmet need for contraception among AGYW in SSA.

## Materials and methods

### Data source

Data was obtained from current DHS conducted between January, 2010 and December, 2018 in 10 SSA countries. These countries were chosen because they are the top 10 countries with high fertility rates in SSA [22]. DHS is a nationwide survey that is carried out across low-and middle-income countries every five-years [27]. The survey focuses on key maternal and child health indicators such as unmet need for contraception. For this study, women’s files (IR) were used and these files contain the responses of women aged 15–49. In selecting the sample for each survey, stratified dual-stage sampling approach was employed. Details of the sampling approach have been described in previous studies [27, 28]. The sample size for this study consisted of AGYW (aged 15–24), who were either married or cohabiting and had complete cases on all the variables of interest (N = 24,898). Table 1 gives detailed information on the survey country, year of survey and sample size for each country.

**Table 1:** Survey and sample size characteristics.

Country	Survey Year	Source of data	Sample (N)	Sample (%)
Angola	2015–16	https://dhsprogram.com/what-we-do/survey/survey-display-477.cfm	2,051	8.24
Burkina Faso	2010	https://dhsprogram.com/what-we-do/survey/survey-display-329.cfm	3,458	13.89
Burundi	2016–17	https://dhsprogram.com/what-we-do/survey/survey-display-463.cfm	1,640	6.59
Chad	2014–15	https://dhsprogram.com/what-we-do/survey/survey-display-465.cfm	3,512	14.11
Gambia	2013	https://dhsprogram.com/what-we-do/survey/survey-display-425.cfm	1,740	6.99
Mali	2018	https://dhsprogram.com/what-we-do/survey/survey-display-517.cfm	2,220	8.92
Mozambique	2015	https://dhsprogram.com/what-we-do/survey/survey-display-467.cfm	1,387	5.57
Niger	2012	https://dhsprogram.com/what-we-do/survey/survey-display-407.cfm	2548	10.23
Nigeria	2018	https://dhsprogram.com/what-we-do/survey/survey-display-528.cfm	3,151	12.66
Uganda	2016	https://dhsprogram.com/what-we-do/survey/survey-display-504.cfm	3,191	12.82
Total	-		24898	100

### Outcome variable

Unmet need for contraception was the outcome variable in this study. To derive this variable, the concept of unmet need for contraception was explained to all the AGYW in order for them to indicate if they had an unmet need for contraception or otherwise. The accompanying responses were; never had sex, unmet need for spacing, unmet need for limiting, using for spacing, using for limiting, no unmet need, not married and no sex in the last 30 days and infecund/menopausal. Following the categorisation of this variable in previous studies [18, 29, 30], AGYW who had never had sex and those who were infecund/menopausal were excluded. To obtain a binary outcome, the rest of the responses were categorised into 0 and 1, where 0 represented ‘no unmet need’ (no unmet need, using contraception for spacing and using for limiting) and 1 for ‘unmet need’ (unmet need for spacing and unmet need for limiting).

### Explanatory variables

Twelve explanatory variables were considered in this study and were grouped into individual level variables and household/community level variables. These variables were not determined a priori; but were selected based on their availability in the dataset, their theoretical relevance and practical significance with unmet need for contraception in previous studies [26, 31–34].

#### Individual level factors

The individual level factors were age, marital status, occupation, educational level, frequency of reading newspaper/magazine, frequency of listening to radio, frequency of watching television and parity. Age was coded as ‘15–19’ and ‘20–24’. Marrital status was recoded into ‘married’, and ‘cohabiting’. ‘Working’ and ‘not working’ were the categories for occupation. Level of education was coded as ‘no education’ ‘primary’ and ‘secondary/higher’. Frequency of reading newspaper/magazine, listening to radio and watching television were each coded as ‘not at all’ ‘less than once a week’ and ‘at least once a week’. Parity was recoded as ‘zero birth’, ‘one birth’, ‘two births’, and three or more births’.

#### Household/Community level factors

The household/community level variables were wealth quintile, sex of household head, place of residence and decision-maker in healthcare. In DHS, wealth quintile is computed using Principal Component Analysis (PCA) [35] and is classified as poorest, poor, middle, richer and richest. Sex of household head was coded as ‘male’ and ‘female’. Place of residence was coded as ‘urban’ and rural’ and decision-maker in healthcare was coded as ‘respondent alone’ and ‘respondent and others’. The selection of wealth quintile, sex of household head, decision-maker in healthcare and place of residence as household/community level variables was based on their categorization in the DHS [27] and in previous studies [36–39].

### Statistical analyses

#### Descriptive analysis

The descriptive analysis involved the use of bar chart to portray the prevalence of unmet need for contraception for each of the 10 countries and chi square test of independence [χ^2^] with 95% confidence intervals (CI) to show the association between unmet need for contraception and the explanatory variables.

#### Modelling approaches

Multilevel logistic regression model (MLRM) was used to examine the association between the individual and household/community factors and unmet need for contraception. The STATA command “melogit” was used in fitting these models. A 2-level model for binary responses was specified, reporting unmet need for contraception or not for AGYW at level 1, in a household/community at level 2. Four models were constructed. The first model was the empty model/null model which is the model that shows the variance in the outcome variable, attributed to the clustering of primary sampling units (PSUs). This model has no explanatory variable. The second model contained only the individual-level factors. Model 3 contained the household/community-level factors. The final model was the complete model that controlled for the individual and household/community factors simultaneously. The selection of variables into the final model was based on their statistical significance at a level of precision of p<0.05.

The MLRM consists of fixed and random effects [40, 41]. The fixed effects (measures of association) shows results of the association between the explanatory variables and the outcome variable and were reported as adjusted odds ratios (aORs) with their 95% CIs, while the random effects (measures of variations) were assessed using Intra-Cluster Correlation (ICC) [42, 43]. LR test was used to check for model adequacy and both Akaike’s Information Criterion (AIC) and Bayesian Information Criteria (BIC) were used to measure how well the different models fitted the data.

To check for correlation among the explanatory variables, a test for multicollinearity was done using the variance inflation factor (VIF) and the results showed no evidence of high collinearity among the explanatory variables (Mean VIF = 1.29, Maximum VIF = 1.57, and Minimum VIF = 1.03**)**. Sample weight (v005/1,000,000) was applied to correct for over-and under-sampling while the SVY command was used to account for the complex survey design and generalizability of the findings.

### Ethical approval

Ethical permissions were not required for this study since DHS datasets which is publicly available was used. Institutions that commissioned, funded, or managed the surveys were responsible for ethical procedures. ICF international as well as an Institutional Review Board (IRB) in each respective country approved all the DHS surveys in line with the U.S. Department of Health and Human Services regulations for the protection of human subjects.

## Results

### Descriptive results

**Fig 1:**
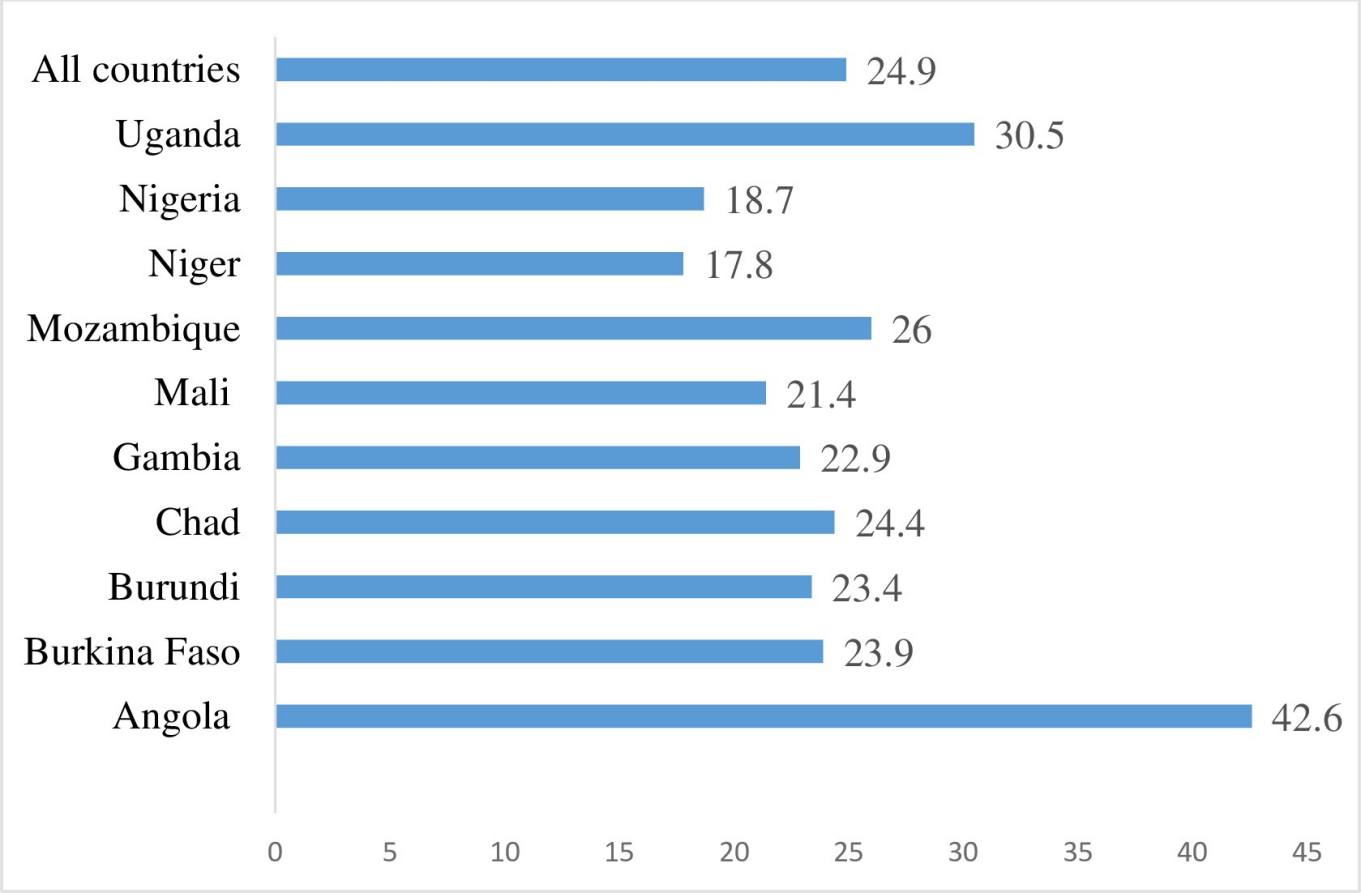
Prevalence of unmet need for contraception among adolescent girls and young women in selected high fertility countries in sub-Saharan Africa.

Fig 1 shows results of the prevalence of unmet need for contraception among AGYW in the 10 selected high fertility countries in SSA. Unmet need for contraception in all the countries was 24.9%, with Angola, recording the highest prevalence of 42.6% while Niger had the lowest prevalence of 17.8%.

Table 2 provides a summary of the distribution of unmet need for contraception among AGYW in the selected high fertility countries in SSA by the explanatory variables. Unmet need for contraception was high among AGYW aged 20–24 (25.7%), those with primary level of education (28.5%), the employed (24.6%), those who were cohabiting (33.0%) and those with three or more births (29.6%). There was also variations in unmet need for contraception in relation to the frequency of reading newspaper/magazine, frequency of listening to radio and frequency of watching television. There were also variations in prevalence of unmet need for contraception across the various household/community factors. AGYW in the middle wealth quintile (25.9%), those in female-headed households (29.5%), those who lived in urban areas (27.6%), and those who took healthcare decisions alone (29.6%) had higher prevalence of unmet need for contraception. Apart from employment status and frequency of listening to radio, all the individual and household/community factors showed significant associations with unmet need for contraception (see Table 2).

**Table 2:** Distribution of unmet need for contraception among adolescent girls and young women by the explanatory variables (Weighted).

Variables	Frequency (n)	Percentage (%)	Unmet need for contraception		p-value
Individual level variables			Yes	No	
**Age**					p<0.001
15–19	7,602	30.5	22.9	77.1	
20–24	17,296	69.5	25.7	74.3	
**Educational Level**					p<0.001
No Education	11,733	47.1	21.7	78.3	
Primary	7,383	29.7	28.5	71.5	
Secondary/Higher	5,781	23.2	26.8	73.2	
**Employment status**					p>0.05
Unemployed	9,583	38.5	24.6	75.4	
Employed	15,315	61.5	25.0	75.0	
**Marital Status**					p<0.001
Married	18,968	76.2	22.3	77.7	
Cohabiting	5,930	23.3	33.0	67.0	
**Frequency of reading newspaper/magazine**					p<0.01
Not at all	22,548	90.6	24.7	75.3	
Less than once a week	1,312	5.3	24.8	75.2	
At least once a weak	1,038	4.2	29.5	70.5	
**Frequency of listening to radio**					p>0.05
Not at all	10,935	43.9	24.9	75.1	
Less than once a week	4,868	19.6	23.6	76.4	
At least once a weak	9.094	36.5	25.4	74.6	
**Frequency of watching television**					p<0.001
Not at all	16,691	67.0	24.0	76.1	
Less than once a week	2,901	11.7	24.6	75.4	
At least once a weak	5,306	21.3	27.8	72.1	
**Parity**					p<0.001
Zero births	4,339	17.4	12.5	87.5	
One birth	8,773	35.2	25.6	74.5	
Two births	6,883	27.7	28.4	71.6	
Three or more births	4,903	19.7	29.6	70.4	
**Household/community level variables**					
**Wealth quintile**					p<0.05
Poorest	5,015	20.1	24.5	75.5	
Poorer	5,726	23.0	25.6	74.4	
Middle	5,317	21.4	25.7	74.1	
Richer	4,838	19.4	25.1	74.9	
Richest	4,001	16.1	23.0	77.0	
**Sex of household head**					p<0.001
Male	21,601	86.8	24.1	75.9	
Female	3,297	13.2	29.5	70.5	
**Place of residence**					p<0.001
Urban	6,206	24.9	27.6	72.4	
Rural	18,692	75.1	23.9	76.1	
**Decision-maker on healthcare**					p<0.001
Respondent Alone	2,565	10.3	29.6	70.4	
Respondent and others	22,333	89.7	24.3	75.7	

### Fixed effects (measures of associations) results

Model 3 is the complete model that shows the associations between the individual and household/community factors and unmet need for contraception among AGYW. With the individual factors, age, educational level, marital status and parity showed associations with the phenomenon. All the household/community level factors showed significant associations with unmet need for contraception among AGYW.

In relation to the individual level factors, the likelihood of unmet need for contraception was low among AGYW aged 20–24 [aOR = 0.82; 95% CI = 0.76–0.88], compared to those aged 15–19. On the contrary, the odds of unmet need for contraception was high among AGYW with primary [aOR = 1.22; 95% CI = 1.13–1.31] and secondary/higher levels of formal education [aOR = 1.18; 95% CI = 1.08–1.28], those cohabiting [aOR = 1.52; 95% CI = 1.42–1.63] and those with three or more births [aOR = 3.41; 95% CI = 3.02–3.85], compared to those with no formal education, those who were married and those with zero births, respectively.

With the household/community level factors, the odds of unmet need for contraception was higher among poorer AGYW [aOR = 1.36; 95% CI = 1.21–1.53], those in female-headed households [aOR = 1.22; 95% CI = 1.13–1.33], urban AGYW [aOR = 1.21; 95% CI = 1.11–1.32] and among AGYW who took healthcare decisions alone [aOR = 1.10; 95% CI = 1.01–1.21], compared to AGYW in the richest wealth quintile, those in male-headed households, those in rural areas and those who took healthcare decisions with others, respectively.

### Random effects (measures of variations) results

As shown in Table 3, in the empty model, there were substantial variations in the likelihood of unmet need for contraception across the clustering of the PSUs (σ2 = 2.0, 95% CI 0.8–5.0). The empty model showed that 6% of the total variance in unmet need for contraception was attributed to between-cluster variation of the characteristics (ICC = 0.06). The between-cluster variations increased by 1% in Model 1, from 6% in the empty model to 7% in the individual-level only model. From Model 1, the ICC declined to 6% (ICC = 0.06) in the community-level only model and remained constant in the complete model (Model 3), which had both the individual and household/community factors. This explains that the variations in the likelihood of unmet need for contraception could be attributed to the differences in the clustering at the primary sampling units (PSUs).

**Table 3:** Multilevel logistic regression models for individual and household/community predictors of unmet need for contraception.

Variables	Model 0	Model 1	Model 2	Model 3
	aOR[95%CI]	aOR[95%CI]	aOR[95%CI]	aOR[95%CI]
**Fixed effects results**				
**Individual level variables**				
**Age**				
15–19		Ref		Ref
20–24		0.81**[0.75–0.87]		0.82***[0.76–0.88]
**Educational Level**				
No Education		Ref		Ref
Primary		1.24***[1.15–1.33]		1.22***[1.14–1.31]
Secondary/Higher		1.21***[1.11–1.32]		1.18***[1.08–1.29]
**Marital status**				
Married		Ref		Ref
Cohabiting		1.59***[1.48–1.71]		1.52***[1.42–1.63]
**Frequency of reading newspaper/magazine**				
Not at all		Ref		Ref
Less than once a week		0.90[0.78–1.03]		0.93[0.81–1.07]
At least once a weak		1.02[0.87–1.20]		1.03[0.88–1.20]
**Frequency of watching television**				
Not at all		Ref		Ref
Less than once a week		1.10[1.00–1.21]		0.91[0.83–1.01]
At least once a weak		1.15***[1.07–1.25]		1.05[0.94–1.18]
**Parity**				
Zero births		Ref		Ref
One birth		2.49***[2.25–2.77]		2.47***[2.23–2.74]
Two births		3.11***[2.78–3.48]		3.07***[2.74–3.43]
Three or more births		3.46***[3.07–3.90]		3.40***[3.02–3.84]
**Household/contextual level variables**				
**Wealth quintile**				
Poorest			1.41***[1.26–1.58]	1.30***[1.15–1.47]
Poorer			1.49***[1.33–1.67]	1.36***[1.21–1.53]
Middle			1.45***[1.29–1.61]	1.33***[1.19–1.49]
Richer			1.33***[1.20–1.48]	1.27***[1.14–1.41]
Richest			Ref	Ref
**Sex of household head**				
Male			Ref	Ref
Female			1.26***[1.16–1.37]	1.22***[1.13–1.33]
**Place of residence**				
Urban			1.42***[1.31–1.54]	1.21***[1.11–1.32]
Rural			Ref	Ref
**Decision-maker on healthcare**				
Respondent Alone			1.24***[1.13–1.35]	1.10*[1.01–1.21]
Respondent and others			Ref	Ref
**Random effects results**				
PSU Variance(95% CI)	2.0(0.8–5.0)	2.2(1.0–5.3)	2.1(0.9–0.51)	2.1(0.9–5.3)
ICC	0.06	0.07	0.06	0.06
LR Test	χ^2^ = 5.38, p< 0.01	χ^2^ = 6.06, p<0.01	χ^2^ = 5.96, p< 0.05	χ^2^ = 5.8, p< 0.01
Wald χ^2^	Reference	778.3***	159.1***	842.1***
Model fitness				
Log-likelihood	-13959	-13523.3	-13880.5	-13490.7
AIC	27922.4	27072.6	27779.1	27019.5
BIC	27938.6	27178.2	27852.2	27181.9
Number of clusters	1159	1159	1159	1159

Exponentiated coefficients; 95% confidence intervals in brackets; AOR adjusted Odds Ratios CI Confidence Interval.

*p< 0.05

**p< 0.01

***p< 0.001.

PSU = Primary Sampling Unit; ICC = Intra-Class Correlation; LR Test = Likelihood ratio Test; AIC = Akaike’s Information Criterion; BIC = Schwarz’s Bayesian Information Criteria.

Model 0 is the null model, a baseline model without any determinant variable.

Model 1 is adjusted for individual level variables.

Model 2 is adjusted for household/community level variables.

Model 3 is the final model adjusted for individual and household/community level variables.

The values of AIC and BIC demonstrated a successive reduction, which shows that there is a substantial improvement in each of the models over the preceding model, and this affirm the goodness of fit of the final model developed in the analysis. Hence, the complete model, which incorporated the individual and household/community factors, was chosen for predicting the occurrence of unmet need for contraception among AGYW.

## Discussion

The purpose of the study was to examine the predictors of unmet need for contraception among AGYW in selected high fertility countries in SSA. This is the first study that has looked at unmet need for contraception among AGYW in selected high fertility SSA countries. It was found that about one-fourth (24.9%) of AGYW in the selected countries had unmet need for contraception, with Angola having the highest prevalence of 42.6%. The study results further showed significant associations between age, level of education, marital status, parity, wealth quintile, sex of household head and decision maker in healthcare and unmet need for contraception.

The prevalence of unmet need for contraception among AGYW in the current study is higher than what has been reported in previous studies in Nigeria [44, 45] but lower than what was found in Burundi [46]. The high prevalence of unmet need for contraception in this study compared to previous studies could be attributed to the different target population and the sample size in the current study and previous studies. While the current study used AGYW in 10 SSA countries, the previous studies were done in individual countries. Interestingly, Angola in this study had the highest prevalence of unmet need for contraception, contrary to what a previous study conducted in the country found [30]. The possible reason for this finding could be that this study focused on AGYW who in previous studies have been found to be more likely to experience unmet need for contraception, compared to older women [30, 46–48].

In this study, it was found that the likelihood of unmet need for contraception was low among AGYW aged 20–24 compared to those aged 15–19. Although previous studies have found all AGYW aged 15–24 to be more likely to have unmet need for contraception, [30, 46–48], the lower odds among older AGYW (20–24) compared to those aged 15–19, could be attributed to barriers to contraceptive access in low-and middle-income countries [49, 50] and the socio-cultural norms surrounding access to contraceptives among adolescents in SSA [51–53]. Again, young women, compared to adolescent girls are more likely to be married and start childbearing and might not have the desire to delay or limit childbearing [54, 55]. Those who have more children may however desire to limit or delay childbearing and can experience unmet need for contraception. This is consistent with our finding that the odds of unmet need for contraception increased with increasing births with AGYW with three or more births having the highest odds of unmet need for contraception compared to those with no births.

AGYW who were cohabiting were more likely to have unmet need for contraception compared to those who were married. This is consistent with previous studies in SSA [30, 46–48]. Some studies have shown that opposition from partners [56, 57] is the reason why cohabiting women have more unmet need for contraception. Other reasons could be that the desire for childbirth is limited during cohabitation, compared to marriage due to socio-cultural norms surrounding childbirth outside of wedlock in most SSA countries [58–60].

AGYW in female-headed households and those who could decide on their own healthcare had higher odds of unmet need for contraception. These findings warrant further study to get deeper explanation. This is because, AGYW in such positions are often considered to be empowered with regards with their health issues, including the decision to use contraceptives [61, 62]. However, it must be added that although AGYW in female-headed households and those who could take their own decisions may have the capacity to consider contraceptive use, the majority of them may still have unmet need for contraception due to barriers to access to reproductive health services among AGYW in SSA, including access and utilisation of contraceptives [63–65].

The study also showed an association between socio-economic status (wealth quintile, level of education and place of residence) and unmet need for contraception. However, there was a disparity in how each of these measures of socio-economic status influenced unmet need for contraception among AGYW. Whereas, higher wealth quintile reduced the likelihood of unmet need for contraception, higher levels of education and living in urban areas increased the likelihood of unmet need for contraception. Specifically with wealth quintile, the study indicated that AGYW in the poorer wealth quintile had higher odds of unmet need for contraception compared with the richest. This association has been observed in previous studies in other SSA countries such as Ghana [48] and Ethiopia [34]. The possible explanation is that AGYW from richer/wealthier households can deal with the cost barrier associated with access to contraceptives, compared to those from poorer households since they can foot both the direct and indirect cost associated with contraceptive uptake [66].

Higher levels of education and urban living increased the likelihood of unmet need for contraception. Similar findings were obtained in Nigeria [44] and Ghana [48]. Just like Solanke, Oyinlola [44] admitted in their study, these findings are counter intuitive. However, the possible reason for these findings could be that AGYW who have higher levels of education and those who live in urban areas are more likely to have higher desire to postpone childbearing in order to fulfil their goals. In this regard, they are more likely to require the use of contraceptives to delay childbearing which they may not get due to the barriers associated with access to contraceptives, particularly stigma. Notwithstanding, these findings warrant further interrogation with a qualitative study.

### Strength and limitations

The use of nationally representative datasets in this current study and the focus on AGYW in high fertility countries in SSA is a major strength in this study. Again, the large sample size and the adoption of well-laid procedures such as training of experienced field enumerators and the use of validated instruments in the DHS strengthen the validity of findings from the dataset. However, the use of cross-sectional design in the surveys makes it impossible to establish causality with respect to the findings. There is also the possibility of response and recall bias since the AGYW may provide social desirable responses and may also find it challenging to recall previous events on unmet need for contraception. Finally, the differences in survey years can limit the comparability of the findings since modernization may have an impact on the prevalence of unmet need for contraception in more current surveys compared to older ones.

## Conclusion

This study has identified disparities in unmet need for contraception among AGYW in high fertility countries in SSA with AGYW in Angola having the highest prevalence. Both individual and household/community level factors predicted unmet need for contraception among AGYW in high fertility countries in SSA. However, based on the ICC values, household/community level factors prevailed the individual level factors. Enhancing access to contraception among poorer AGYW, those in female-headed households, those in urban areas and those who take healthcare decisions alone by both governmental and non-governmental organisations in high fertility countries is recommended.
